# Hospital Medical Schools and the King's Fund

**Published:** 1910-12-03

**Authors:** 


### HOSPITAL MEDICAL SCHOOLS AND THE KING'S FUND.

The Metropolitan Radical Federation has addressed the following letter to the Secretaries of the King's Fund in consequence of the correspondence we published last week on pp. 265-66 of The Hospital :—

November 25, 1910.

Gentlemen,—Your letter of November 18 has been laid before the Metropolitan Radical Federation, and I am directed to make the following reply to it :—

You seem to have failed to observe that it was in 1908, on October 3, and not in 1909, that Dr. Charles Slater in a reported speech at St. George's Medical School flouted the report of Sir Edward Fry's Commission, and spoke of the "ill-advised action of a Commission of the King's Hospital Fund.".

This insubordinate utterance foreshadowed most plainly an intention at St. George's of disregarding the report of Sir Edward Fry's Commission, and it should, in the opinion of the Metropolitan Radical Federation, have placed the King's Fund on its guard against making any grant to St. George's Hospital, after that speech was delivered, until some clear undertaking had been made by the hospital authorities that this verbal flouting of the Commission would not be followed and endorsed by an actual violation of its findings.

In default of any such precaution on the part of the King's Fund that violation actually took place in 1909, and the request of the Metropolitan Radical Federation to his Majesty was, and is, that no more grants shall be made to St. George's Hospital from the King's Fund until the £1,576 diverted in 1909 to the school from the general fund of the hospital are refunded.

This request remains upon record, and the Metropolitan Radical Federation will await with patience the decision of the King's Fund as to whether it will comply with it or not.

With regard to the enormous sums paid over to medical schools from hospital funds previously to the report of the Commission, the Metropolitan Radical Federation learn with great regret that no effort will ever now be made to ascertain how much of them were in the minds of the Commissioners when they spoke of the schools remaining debtors to the hospitals.

The Metropolitan Radical Federation, in their letter of October 31, drew attention to the fact that the King's Fund, collected and subscribed for the benefit of the poor and the working classes, is managed by a committee not elected by the subscribers, and comprising no single representative of those classes for which the great hospitals were founded and are maintained, and they observe with regret that your letter makes no other response to this paragraph than a reference to an Act of Parliament. If that Act precludes the working classes from any participation in the management of the King's Fund the Metropolitan Radical Federation are of opinion that it should be amended.

In conclusion, the Metropolitan Radical Federation beg leave to point out that the King's Fund was raised, and still appeals for subscriptions, in the name of his Gracious Majesty, and for that reason they are of opinion that the King himself is the person to whom they should appeal for the redress of such errors in its administration, as they have pointed out, and not to the Executive Committee, whose management of the fund in certain particulars they have deemed it their duty to impugn.

Your obedient servant,

E. Garrity.

THE KING'S LETTER.

In connection with the foregoing correspondence Mr. Garrity writes to us as follows :—

37 Hall Street, City Road, E.C.
November 26, 1910.

Sir,—I observe that the Hon. Secretaries of King Edward's Hospital Fund, in sending you the letter addressed by the Metropolitan Radical Federation to the King, omitted to accompany it with His Majesty's reply. The correspondence therefore as published by you will lead the public to believe that the King made no response to our personal appeal to him, but merely handed our letter over to the Hon. Secretaries of King Edward's Hospital Fund without comment.

As a matter of fact the King, by the hand of his Secretary, wrote us a most considerate reply, which was as follows :—

"Dear Sir,—In reply to the communication which you forwarded to the King on the 31st October, from the Metropolitan Radical Federation, I am commanded to inform you that His Majesty fully appreciates the interest evinced by this letter on the part of the Federation in the work and administration of the London Hospitals. But His Majesty is no longer President of King Edward's Hospital Fund. There is, therefore, no course open to me but to forward the correspondence to the Hon. Secretaries of the Fund, by whom, I feel sure, careful attention will be paid to the subject with which it deals.—Yours very faithfully, (sgd.) Arthur Bigge."

Your obedient servant,

Edw. Garrity.

The Editor, The Hospital.

